# MicroRNAs as Regulators of Reproductive Function: From Gametogenesis to Pregnancy

**DOI:** 10.3390/jdb14030038

**Published:** 2026-09-01

**Authors:** Sabrina Roy, Karina Gutierrez, Vilceu Bordignon, Werner Giehl Glanzner

**Affiliations:** Department of Animal Science, McGill University, Macdonald Campus, Sainte Anne de Bellevue, QC H9X3V9, Canada; sabrina.roy2@mail.mcgill.ca (S.R.); karina.gutierrez@mcgill.ca (K.G.); vilceu.bordignon@mcgill.ca (V.B.)

**Keywords:** miRNAs, reproductive biology, embryo development, sperm, embryo genome activation, pregnancy

## Abstract

The regulation of transcriptional activity constitutes a primary layer of control over cellular function across diverse tissues and cell types. This regulatory transcriptional landscape is largely governed by epigenetic mechanisms, including DNA and histone modifications, the dynamic activity of epigenetic “writers” and “erasers,” and the influence of small regulatory molecules such as microRNAs (miRNAs). miRNAs are small non-coding RNA molecules that play critical roles in transcriptional and post-transcriptional gene regulation. By modulating the expression of transcription factors, kinases, and other regulatory proteins, miRNAs exert tight control over cellular processes and maintain homeostasis. Given the essential requirement for precise cellular coordination in reproductive biology, miRNAs have emerged as key regulators of reproductive function. They participate in multiple stages of reproductive processes, ranging from ovarian function to the establishment and maintenance of pregnancy, thereby influencing fertility outcomes across species. In this review, we summarize recent advances in understanding the roles of miRNAs in key reproductive events, including gametogenesis, oocyte maturation, embryonic genome activation, early embryonic development, implantation and pregnancy. We aim to highlight the principal miRNAs involved in the maintenance of reproductive function and fertility, and their molecular targets and the regulatory networks when known. Based on our review, we conclude that although their potential in clinical and/or agricultural applications still remains uncertain, their role in regulating fundamental aspects of cellular biology, particularly reproductive processes, is becoming increasingly clear.

## 1. Introduction

The central dogma of molecular biology states that genetic information is typically processed from DNA to RNA to protein, and that this flow ultimately determines cellular and organismal phenotype [1]. However, over the past few years, it has become evident that not all RNA molecules proceed to protein translation. Large-scale projects aimed at the systematic annotation and functional characterization of genes, such as ENCODE and FANTOM, have revealed that at least 80% of mammalian genomic DNA is actively transcribed and tightly regulated, with the vast majority of these transcripts classified as non-coding RNAs (ncRNAs) [2,3] that do not encode proteins. This discovery demonstrates that many regulatory RNAs function independently of protein translation, prompting a re-evaluation of the roles of RNA in the development and evolution of higher organisms [4]. Although ncRNAs were originally considered transcriptional background with little biological significance, the identification of ribosomal RNA (rRNA) and transfer RNAs (tRNAs) in the late 1950s marked the beginning of a new field of study that greatly expanded our understanding of epigenetic regulation and development. Non-coding RNAs are now broadly categorized into housekeeping RNAs, which include rRNAs, tRNAs, and small nuclear RNAs (snRNAs), and regulatory RNAs, such as microRNAs (miRNAs) and small interfering RNAs (siRNAs) [5].

Among these ncRNAs, miRNAs have been increasingly highlighted for their critical roles in disease, fertility, and development through post-transcriptional regulation of gene expression [6]. miRNAs are a class of small (19–24 nucleotides), endogenous, evolutionarily conserved RNAs that function by binding complementary sequences in messenger RNA (mRNA), thereby interfering with the translational machinery and modulating protein production [7]. First discovered in 1993 by Lee and colleagues [8], miRNAs have since become a major focus of research due to their widespread regulatory roles. Notably, many miRNAs are highly conserved across species [9], which is often associated with the preservation of similar functions. For example, miR-1 exhibits muscle-specific expression in nematodes, fruit flies, and humans [10,11,12], suggesting an essential and evolutionarily conserved role in muscle development across the animal kingdom [9]. Another important feature of miRNAs is their potential utility as biomarkers. miRNAs are commonly associated with extracellular vesicles (EVs), such as exosomes [13] or with proteins such as AGO2, both of which are thought to protect them from degradation and enhance their stability in the extracellular environment [14,15]. This stability allows miRNAs to persist in biological fluids such as serum [16,17], follicular fluid [18], uterine fluid [19], and seminal plasma [20], making them particularly attractive candidates for non-invasive disease detection and monitoring [21,22]. When the regulatory roles and target genes of specific miRNAs are understood, their expression profiles can be leveraged to aid disease diagnosis, monitor gene regulation, and assess disease progression. In addition to their diagnostic potential, miRNAs can be synthesized relatively easily and inexpensively in vitro as miRNA mimics, making them standard and versatile tools in research with emerging therapeutic potential [23].

While miRNAs are best known for their post-transcriptional regulation of gene expression in the cytoplasm, more recent studies have revealed that they can also localize to the nucleus. Within the nucleus, miRNAs can target gene promoters or enhancers, facilitate the nuclear translocation of mRNAs, and regulate signalling pathways by binding to specific protein receptors [24,25,26]. Through these diverse mechanisms, miRNAs contribute to the regulation of gene expression as well as transcriptional and translational activity. Notably, these mechanisms allow miRNAs to regulate numerous biological and physiological processes, including cell fate and differentiation [27], cell cycle and proliferation [28,29], apoptosis [30], and developmental timing [31]. Consequently, gaining a deeper understanding of miRNAs’ function and their gene targets has significant implications for advancing our understanding of biological regulation and its impact on developmental and physiological outcomes.

## 2. Global Roles of miRNAs in Development and Reproductive Biology

In general, miRNAs are involved in fundamental cellular processes, including cell growth, differentiation, and programmed cell death [32]. Due to these broad regulatory effects, miRNAs influence many physiological and pathological processes throughout the body. One of the most critical roles of miRNAs is in development, particularly within reproductive biology. Numerous studies have shown that animals lacking miRNA expression fail to survive or reproduce normally [33,34]. Global disruption of the miRNA pathway through DICER knockdown in fruit flies and mice results in embryonic lethality, widespread abnormal organ morphology, and a significant depletion of stem cell populations [33,34,35,36,37,38]. Embryonic stem cell differentiation, a central event in organ and organ system development, is strongly regulated by miRNAs [7,39]. In mice, although DICER deficiency does not prevent the formation of embryonic stem cell colonies, it severely compromises their differentiation capacity and leads to pronounced defects in cellular morphology [40].

Beyond stem cell regulation, miRNAs play crucial roles across multiple stages of mammalian reproductive development, including sex differentiation [41], gametogenesis [42], fertilization [43], embryo genome activation and early embryonic development [44], implantation [45], germ layer specification [46], and pregnancy [47,48]. Together, this underscores the central and multifaceted role of miRNAs in regulating normal development and reproductive success.

Although miRNAs are critically important, relatively little is known about the specific gene targets and precise functions of individual miRNAs in reproductive processes in mammalian species. Therefore, this review summarizes some of the current evidence of miRNAs’ roles in reproduction (Table 1), specifically from gametogenesis through early embryonic development and pregnancy, highlighting both their developmental importance and the key areas where further research is needed.

## 3. Mirnas in Male Gametogenesis and Sperm Function

Spermatogenesis is an essential process for male fertility that involves immature germ cells (i.e., spermatogonia) in the testes, which undergo mitotic and meiotic divisions, followed by differentiation, to form mature, motile spermatozoa. This process is regulated by both endocrine systems, through several hormones produced in different tissues that target reproductive organs, and by local autocrine regulation, with testicular cells playing a major role [49,50,51]. Moreover, spermatogenesis depends not only on this tight regulation and stage-specific expression of protein-coding genes but also on a broad array of non-coding RNAs, including miRNAs [52,53]. Despite their recognized importance, the precise roles of miRNAs in male gonadal development remain incompletely understood.

To elucidate the role of miRNAs in spermatogenesis, studies have explored miRNA expression patterns and correlated them with the expression of target genes encoding proteins involved in sperm development, structure, motility, and metabolism. Results from some of these studies reported increased expression of let-7a, -7d, -7e, and miR-22 in abnormal samples, alongside decreased expression of miR-15b compared to normal control samples [54] in pig sperm. Although the precise roles of these miRNAs in sperm function remain unclear, bioinformatic analyses revealed that the mRNA targets of these differentially expressed miRNAs encode proteins previously implicated in sperm function [54]. Still in pigs, specific miRNAs have been found in higher levels in sperm samples of breeding boars with higher fertility after artificial insemination [55,56]. In other large animal species, such as bovine [57], miRNAs have been directly linked to fertility outcomes. In this context, spermatozoa appear to carry a complex molecular cargo, including intracellular miRNAs that can be transcribed and modulate key molecular functions, as well as miRNAs secreted via sperm-derived extracellular vesicles, which play an important role in molecular regulation processes [52,58].

A particularly important miRNA family, miR-34/449, has been shown to play crucial roles in normal testicular function, successful spermatogenesis, and the regulation of spermatozoa maturation and function across multiple species. Accordingly, abnormal expression of miR-34/449 family members contributes to the development of oligoasthenoteratozoospermia and male infertility [59]. The importance of this miRNA family extends beyond spermatogenesis, as miR-34c has been identified as a sperm-born miRNA with potential roles in fertilization and early embryonic development. In mice, inhibition of sperm-borne miR-34c activity resulted in reduced first cleavage division and suppressed DNA synthesis in zygotes, highlighting its importance during early embryogenesis [60].

Clinical studies in humans further support the importance of miR-34 family members in male fertility. Analysis of sperm samples from 102 infertile and 52 fertile men showed that miR-34a, miR-34b, and miR-34c expression levels were significantly lower in infertile men than in fertile controls [61]. In addition, men with low miR-34c-5p expression exhibited reduced sperm motility despite having normal sperm morphology [62]. A subsequent study involving 106 couples undergoing ICSI examined the relationships among sperm DNA fragmentation (SDF), miR-34c-5p, miR-449b-5p, fertilization, and embryo development [63]. SDF was negatively correlated with sperm concentration, progressive motility, normal morphology, and the number of viable embryos, confirming the detrimental effects of DNA damage on embryo development. Interestingly, SDF was positively correlated with both mir-34c-5p and mir-449b-5p, suggesting that these miRNAs may influence sperm chromatin integrity through epigenetic mechanisms [63]. However, while increased mir-34c-5p expression was associated with a greater likelihood of obtaining viable embryos, elevated mir-449b-5p expression increased the risk of embryo developmental failure [63].

The relationship between sperm miR-34c and embryo developmental competence has also been demonstrated in assisted reproductive technology (ART). Analysis of 412 oocytes submitted to fertilization using sperm samples obtained from 58 men with reported infertility revealed that miR-34c was absent in unfertilized oocytes but present in zygotes with three pronuclei, arrested embryos at days 3 and 6, blastocysts and normal day 3 embryos, while its levels were positively correlated with blastocyst formation, high-quality blastocyst rates, and pregnancy outcomes [64]. These findings suggest that sperm miR-34c may serve as a clinically relevant biomarker for predicting blastocyst development, embryo quality, and pregnancy success.

More recently, altered expression of miR-23a-3p and miR-23b-3p has also been associated with impaired male fertility in humans [65,66]. Both miRNAs were found to be significantly upregulated in patients with oligoasthenozoospermia, with their expression levels showing an inverse correlation with sperm count, sperm motility, and the proportion of morphologically normal spermatozoa [67]. Bioinformatic analyses further identified four spermatogenesis-related target genes of miR-23a/b-3p, including HMMR (hyaluronan-mediated motility receptor), PFKFB4 (6-phosphofructo-2-kinase/fructose-2,6-bisphosphatase 4), SPATA6 (spermatogenesis-associated protein 6), and TEX15 (testis-expressed protein 15), all of which are involved in maintaining normal sperm quality and spermatogenesis [67]. Consequently, increased expression of miR-23a/b-3p may impair spermatogenesis through the repression of these key regulatory genes [68].

There is growing evidence that sperm miRNAs present at fertilization may also influence the health and development of future offspring. Increased evidence suggests that paternal exercise can influence the cardiometabolic health of offspring, with sperm miRNAs believed to play a central role in this process [69]. Along the same lines, it has been observed that male mice fed a high-fat diet produced offspring that developed impaired glucose tolerance, increased adiposity, and reduced skeletal muscle glucose uptake [69,70]. However, when paternal exercise was introduced prior to conception, the offspring exhibited improved glucose homeostasis, enhanced endurance capacity, and increased skeletal muscle mitochondrial biogenesis, particularly in male progeny [69,70]. Supporting these findings, an 8-week low-fat diet combined with exercise altered sperm miRNA expression profiles, particularly X-linked miRNAs, which are considered strong candidates for mediating epigenetic inheritance [69,71]. Collectively, these findings further highlight the importance of miRNAs in sperm biology, demonstrating that their roles extend beyond regulating spermatogenesis, morphology, and sperm quality. The miRNA composition of sperm itself appears to be critical for shaping the sperm’s contribution to the developing embryo and may ultimately influence offspring health and development. However, further research is still required to achieve a comprehensive understanding of the underlying molecular and epigenetic mechanisms regulating proper sperm production. Such advances are essential to determine whether sperm RNAs, particularly sncRNAs, are true drivers of male fertility [72].

## 4. Mirnas in Oogenesis, Folliculogenesis and Oocyte Maturation

Oogenesis, analogous to spermatogenesis, is the developmental process in females that gives rise to the female gametes (i.e., oocytes) within the ovaries. This process begins during fetal development with the formation of primary oocytes that remain quiescent until puberty. Under hormonal regulation, oocyte maturation resumes cyclically, continuing until menopause, typically resulting in the ovulation of a single mature oocyte per reproductive cycle, which becomes available for fertilization [73,74]. Successful oogenesis is crucial for producing a developmentally competent oocyte capable of supporting fertilization and early embryonic development. Because the oocyte must initiate and sustain early developmental processes before embryonic genome activation (EGA), oogenesis involves the accumulation of cytoplasmic enzymes, mRNAs, organelles, and metabolic substrates necessary for metabolism and development [75]. This extensive maternal contribution also suggests an important role for regulatory molecules such as miRNAs during oocyte development.

Supporting this idea, studies have investigated the role of miRNAs in the follicular environment, including cumulus cells, follicular fluid, and oocytes during oogenesis and oocyte maturation. In pigs, miR-378 has been shown to influence oocyte maturation in vitro [76]. miR-378 levels decrease in cumulus cells as oocytes mature from the germinal vesicle (GV) to metaphase II (MII) stage, indicating a regulatory role during cumulus-oocyte complex (COC) maturation [76]. Nevertheless, overexpression of miR-378 impaired cumulus expansion, reduced expression of genes involved in expansion and oocyte maturation, suppressed meiotic progression, and decreased blastocyst formation following fertilization [76]. These effects were associated with reduced aromatase (CYP19A1) expression and decreased estradiol production, as supplementation reversed the inhibitory effects of miR-378, suggesting that miR-378 regulates COC maturation in part by suppressing estradiol synthesis [76]. Similarly, in ovine granulosa cells, miR-27a-3p has been shown to regulate estradiol synthesis by directly inhibiting CYP19A1 expression, further highlighting the importance of miRNA-mediated regulation of aromatase activity in follicular development [77]. In mice, miR-21 was shown to regulate oocyte maturation, mainly acting through the cumulus cells, primarily by modulating glutathione levels and targeting the Bmpr2 and Ptx3 genes [78]. These findings demonstrate that miRNAs, such as miR-378, miR-27a-3p and miR-21, can modulate gene expression and function in cumulus cells, thereby affecting oocyte maturation through oocyte-cumulus communication and paracrine regulation.

Similarly, miR-101 has been identified as a regulator of oocyte maturation in vitro by targeting hyaluronic acid synthase 2 (HAS2) in cumulus cells [79]. On top of that, comparative miRNA profiling of cumulus cells from GV and MII oocytes revealed miR-101 as a key differentially expressed miRNA, and functional analyses demonstrated that its mediated suppression of HAS2 inhibits cumulus expansion and impairs oocyte maturation [79]. Besides miR-101, miR-31 [80] also impacts oocyte maturation through regulation of cumulus expansion by modulation of the MAPK signalling pathway, while miR-125a targets maturation-related target genes, specifically the adenosine deaminase RNA-specific (ADAR) [81], responsible for modulating several reproductive processes from embryo development to implantation. The role of miRNAs, more specifically miR-29b, in oocyte maturation is not restricted to targeting genes related to cumulus expansion and meiotic progression, but also to targeting metabolism-related genes, such as CYS, the gene encoding cytochrome C, resulting in increased ROS and consequent lower levels of oocyte maturation [82].

miRNAs regulate multiple aspects of folliculogenesis through the regulation of granulosa cell proliferation, follicular development, and oocyte maturation. miR-181a negatively regulates granulosa cell proliferation by directly targeting activin receptor IIA (ACVR2A), thereby reducing the expression of cyclin D2 and proliferating cell nuclear antigen (PCNA) and inhibiting activin-Smad2 signalling [83]. Conversely, overexpression of ACVR2A counteracted the inhibitory effects of miR-181a, confirming this regulatory interaction [83]. Similarly, miR-184 promotes follicular development by exerting anti-atretic effects through the induction of SMAD3 expression after being transcriptionally activated by SREBF2 in an H3K4me3-dependent manner [84]. Given that more than 99% of ovarian follicles undergo apoptosis [85], studies have investigated miRNAs’ involvement in this process. Comprehensive mechanistic analyses demonstrated that miR-184 regulates granulosa cell survival and follicular atresia through multiple complementary pathways, highlighting its potential as a promising regulator of follicular development [84]. To further investigate miRNAs’ involvement in follicular atresia, miRNA expression profiling has been done in healthy, early atretic, and progressively atretic follicles [86]. Among the differentially expressed miRNAs identified, miR-26b was upregulated during follicular atresia and was shown to promote granulosa cell apoptosis by increasing DNA breaks through direct targeting of the Ataxia telangiectasia mutated (ATM) gene in vitro [86]. Besides follicular atresia, ovarian reserve is also impacted by miRNA regulation, more specifically miR-330-5p, in combination with lncRNAs and histone deacetylases (HDAC3) [87]. Beyond regulating ovarian reserve, miRNAs have also been implicated in follicular dominance. For example, oar-miR-143 was shown to play a key role in follicular dominance in prolific sheep by targeting and regulating CDK2 expression [88].

Collectively, these studies illustrate that miRNAs play diverse and critical roles throughout oogenesis, influencing follicular development, oocyte maturation, follicular atresia, and ovarian reserve, underscoring their essential contribution to the earliest stages of reproduction.

## 5. Mirnas During Embryo Genome Activation and Early Development

EGA is the critical developmental process that occurs following fertilization, during which the embryo transitions from relying on maternally deposited transcripts to initiating its own transcriptional programme [89,90,91]. During this transition, several key regulatory mechanisms occur, including maternal message clearance, chromatin remodelling, and transcriptional initiation, all of which are essential for proper embryonic development and viability [92,93]. Moreover, maternal transcript clearance is a crucial step for the normal progression of EGA, since it must be selectively degraded [94], and miRNAs have been hypothesized to play an important regulatory role in this specific step. In non-mammalian model organisms such as zebrafish (*Danio rerio*), African clawed frog (*Xenopus laevis*), and common fruit fly (*Drosophila melanogaster*), miRNAs are already known to be required for the clearance of maternal products, a process that is crucial for successful embryogenesis [95,96,97,98]. However, the role of miRNAs during EGA in mammalian species remains less well characterized. In bovine embryos, in which EGA takes place around the 8-cell stage [99], some studies have reported that the relative abundance of miRNA increases during the 8-cell stage, corresponding to the EGA expected timeframe [100]. Nevertheless, other reports have shown that the miRNA molecular machinery is present throughout preimplantation development, from the 1-cell stage to the blastocyst [44]. Additionally, researchers distinguished maternal from embryonic miRNAs by culturing embryos in the presence of α-amanitin, revealing that embryonic miRNA expression begins at the two-cell stage and increases substantially at the morula and blastocyst stages [44]. Furthermore, disruption of miRNA biogenesis via DGCR8 knockdown significantly reduced embryonic miRNA expression, blocking the transition from morula to blastocyst, resulting in an accumulation of mRNAs predicted to be targets of embryonic miRNAs in DGCR8-deficient embryos [44]. In bovine, recent evidence indicates that dysregulation of miR-21 and/or exposure to bisphenol during oocyte maturation affects embryonic development, resulting in arrested 8-cell embryos and blastocysts [101]. Similarly, miR-146b has been shown to negatively regulate embryo development, since its overexpression was observed in embryos that fail to develop in comparison to embryos that reach the blastocyst stage, while its inhibition reduced apoptosis [102]. In the same study, EVs secreted by these non-blastocysts were also enriched for miR-146b. Moreover, studies highlight a potential effect of in vitro culture conditions, such as oxygen tension, on the miRNA cargo secreted by embryos [103].

In other aquatic species, such as zebrafish, hybrid fish and medaka, miRNAs also participate in EGA and embryo development [104,105,106], with miR-430 being an important regulator of EGA in zebrafish and hybrid fish [104,105] but having only a post-EGA role in medaka [106]. In pigs, the role of miRNAs in EGA remains unclear. Recent evidence has highlighted a possible role for miR-200a-5p, as it was shown to be upregulated during the expected EGA period [107]. More mechanistic studies in pig embryos have investigated the relationship between miR-21 and its target programmed cell death 4 (PDCD4) during porcine oocyte in vitro maturation and assessed the consequences of miR-21 inhibition on early embryonic development [108]. PDCD4 is a crucial tumour suppressor protein that inhibits cell growth and promotes programmed cell death by blocking protein synthesis and interfering with cancer-promoting signalling pathways [109,110,111]. The authors demonstrated that miR-21 is differentially expressed during meiotic maturation and that its inhibition alters PDCD4 protein abundance. Interestingly, inhibition of miR-21 activity during in vitro maturation with antisense miR-21 reduced embryo development to the 4–8-cell stage following parthenogenetic activation. This suggests post-transcriptional regulation by miR-21 can influence subsequent embryonic development [108]. Moreover, other miRNAs such as miR-155 have been shown to negatively affect embryonic development in porcine embryos once its inhibition resulted in enhanced preimplantation development together with downregulation of stress- and apoptosis-related genes by together with downregulation of P53 and TNF-α [112].

Studies focusing on later stages of embryonic development compared the profiles of EVs from in vivo- and in vitro-derived bovine embryos during blastulation and hatching. They found that in vivo embryos exhibited greater developmental competence and secreted larger, more stable EVs than in vitro embryos [113]. In vitro embryos not only produced smaller and more abundant EVs but did so especially during the hatching stage, suggesting that EV production can be influenced by both embryo origin and developmental stage [113]. Additionally, in vivo-derived EVs were enriched in miRNAs involved in implantation and cell lineage specification, including miR-124, miR-125, and miR-181, whereas in vitro-derived EVs contained higher levels of stress and apoptosis-associated miRNAs such as miR-23b, miR-92a, and miR-409 [113]. Similarly, previous studies have suggested that EV secretion and miRNA abundance are regulated by embryonic development and that the miRNA cargo participates in regulating key pathways important for embryonic development, implantation, and pregnancy [114]. This was further explored when embryos were cultured with supplementation of EVs isolated from follicular fluid, and metabolic and developmental outcomes were partially modified in the resulting embryos [115]. Together, these studies suggest that embryonic origin and developmental stage shape distinct EV-mediated regulatory programmes. These findings altogether emphasize the critical role of miRNAs during EGA and early development and underscore the need for continued research across species to understand their functions better and translate this knowledge into in vivo applications, with potential use in clinical trials and/or breeding programmes.

## 6. Mirnas in Embryo Implantation and Pregnancy

Taken together, the biomarker potential of miRNAs, along with growing knowledge of their roles in embryo–uterus communication and embryo implantation, makes studying fetal development and pregnancy a logical next step for assessing how effectively miRNA regulation can predict fetal health and developmental outcomes. Studies have shown that the endometrium responds to the embryo’s EV miRNA cargo, resulting in viable or nonviable pregnancy [116]. Furthermore, secreted EVs containing miRNAs can mediate communication between embryos, the uterus, and the corpus luteum, facilitating interactions that support and maintain pregnancy viability [117]. This observation aligns with reported differences in the miRNA cargo of EVs isolated from the oviductal fluid of pregnant cows [19,118]. These EV-coupled miRNAs have been shown to modulate the metabolic profile of embryos, thereby impacting their development [119]. More specifically, miR-124-3p has been shown to negatively affect implantation by reducing uterine receptivity [120]. Moreover, a low abundance of pregnancy-related miRNAs is related to impaired pregnancy establishment in cloned embryos in bovine [121]. On the other hand, some miRNAs, such as miR-17-5p, secreted from oviductal fluid, are positively correlated with embryonic development [122], possibly regulating embryo–uterus crosstalk and thereby increasing pregnancy rates. As has been shown in bovine models, circulating miRNAs in pigs play an important role in embryo-maternal communication and therefore critically participate in the implantation process [123]. This becomes evident in studies reporting differences in miRNA abundance between serum from pregnant and non-pregnant animals [124]. Researchers characterized several reproductive status-dependent miRNAs in serum samples collected from both estrous-cycling and pregnant pigs. They found that serum samples were enriched for miRNAs associated with key reproductive processes, including cell viability, angiogenesis, and embryonic development, during the estrous cycle and early pregnancy [124]. Further analysis showed that levels of ssc-miR-143-3p and ssc-miR-125b differed between pregnant and non-pregnant animals, with ssc-miR-125b also correlating positively with litter size [124]. Similarly, genetic variants in the ovine miR-27a gene were significantly associated with litter size, further supporting the role of miRNAs as important regulators of reproductive performance and prolificacy across livestock species [77]. Nevertheless, a balance among the appropriate miRNAs is necessary to regulate trophoblast cell proliferation and pregnancy outcomes. In this context, while miR-26a-5p stimulates trophoblast proliferation, miR-125b-5p has been shown to decrease trophoblast cell migration [125,126], impacting pregnancy outcomes in pigs. Other miRNAs, such as miR-192, have been suggested as early pregnancy biomarkers in pigs [127] due to their role in targeting ITGA4, an important factor in embryo implantation. The role of miRNAs during later stages of pregnancy is not only restricted to implantation and successful pregnancy, as it has been shown that the dietary composition of pregnant sows influences the miRNA profile of their offspring [128,129].

During pregnancy, miRNAs are described to have a role in preeclampsia [130,131], mostly in humans. Preeclampsia is a pregnancy-related disorder characterized by maternal hypertension, often accompanied by organ damage, and currently has no definitive treatment aside from preventive measures for high-risk patients or delivery of the fetus. The condition affects an estimated 2–8% of pregnancies worldwide and remains one of the leading causes of maternal and perinatal morbidity and mortality [132,133]. In the context of miRNA and preeclampsia, several miRNAs have been investigated for their potential roles in the condition. For instance, miR-210 has been shown to play an important role as a potential early biomarker for preeclampsia [134], since serum miR-210 expression was significantly associated with a diagnosis of preeclampsia, while its overexpression reduced trophoblast invasion, a process essential for proper uteroplacental perfusion and healthy placental development [134], highlighting its potential as a novel non-invasive biomarker for early detection. Besides miR-210, both miR-21 [135] and miR-17-3p [136] have been shown to be altered in patients with preeclampsia compared with normal pregnancy, with the latter responsible for inducing oxidative stress in tissues. Furthermore, miR-214-3p has been suggested as a preventive target for preeclampsia [137]. Finally, understanding the role of miRNAs in this condition would facilitate early identification, enable closer monitoring, earlier preventive interventions, and improve management, potentially reducing maternal and fetal complications.

Overall, findings that correlate miRNAs with embryo-uterine communication, implantation, and pregnancy maintenance further reinforce the importance of miRNAs in reproductive biology and highlight their potential as indicators of reproductive status and health, as well as promising tools for future applications in agricultural breeding programmes.

## 7. Outstanding Questions and Future Perspectives

**Figure 1:**
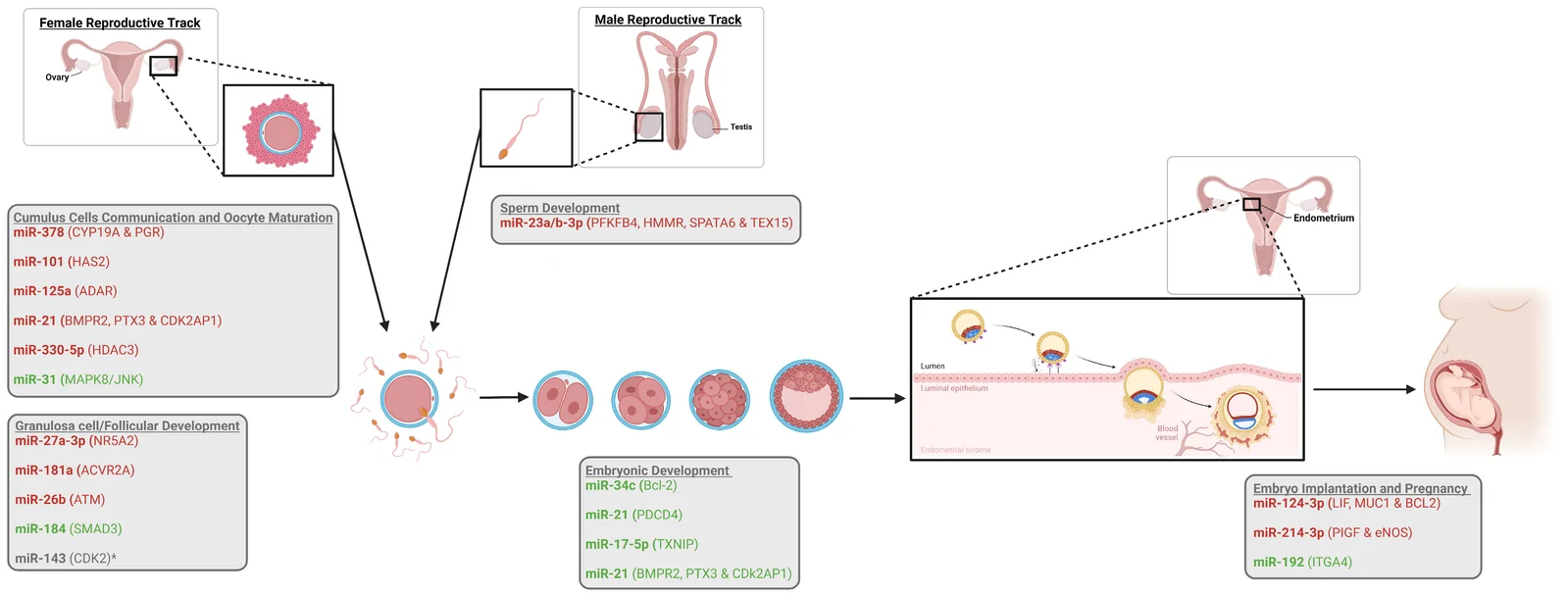
Selected miRNAs and their targets across key reproductive processes in various mammalian species. Only representative miRNAs with identified targets are included. Targets are shown in parentheses for each corresponding miRNA. miRNAs associated with positive phenotypes/outcomes are shown in green, while those correlated with negative phenotypes/outcomes are shown in red. * Possibly related to a positive phenotype but lacking functional validation. Created in BioRender. Bordignon, V. (https://BioRender.com/4nv9jy2).

Based on the findings of this review, miRNAs emerge as highly promising regulatory molecules with substantial potential for diagnostic applications and for modulating transcriptional activity through specific targets during crucial reproductive processes (Figure 1). Although numerous studies have explored the roles of specific miRNAs in mammalian species and many findings are encouraging, considerable gaps in knowledge remain, particularly regarding the precise gene targets and stage-specific functions of individual miRNAs, as highlighted in Table 1.

Much of the current understanding of miRNA functions in reproduction is derived from expression profiling and bioinformatic predictions, highlighting the need for functional studies that directly link specific miRNAs to defined molecular pathways and developmental outcomes. In this context, miRNA sequencing technologies, combined with systematic identification and validation of target genes, represent crucial next steps toward deepening our understanding of miRNA-driven regulatory networks.

Another important consideration for the future of miRNA research in reproduction is how the growing body of knowledge can be translated into practical applications. Numerous studies have identified miRNAs associated with reproductive traits or disorders; for example, reduced expression of the miR-34 family has consistently been linked to impaired male fertility and decreased sperm motility [61,62]. miR-184 has been highlighted as a promising regulator of follicular development because of its anti-atretic effects in female reproduction [84]. Identifying these associations is only the first step; however, how this knowledge can be applied in specific clinical or agricultural systems remains to be determined. Furthermore, the feasibility of inducing or inhibiting specific pathways in vivo through the modulation of single or multiple miRNAs also remains unclear. In this context, future research should focus on determining how this knowledge can be leveraged to improve fertility outcomes, whether through the development of diagnostic biomarkers, therapeutic interventions, or strategies to enhance reproductive technologies.

Importantly, this growing scientific interest is now being mirrored by major investments from the pharmaceutical and biotechnology sectors, highlighting the perceived translational potential of miRNA-based therapies. For example, recent high-value acquisitions such as Novartis’s $800 million purchase of Regulus Therapeutics for its renal disease candidate farabursen, and Novo Nordisk’s $1 billion acquisition of Cardior Pharmaceuticals targeting microRNA-132 in heart failure [138], underscore increasing confidence in RNA-based therapeutic strategies [139]. However, despite this momentum, miRNA therapeutics remain in relatively early stages of development compared to other RNA modalities, with only a limited number advancing into clinical trials and none yet reaching phase III [139]. This gap between strong commercial interest and limited clinical maturity highlights both the promise and the complexity of miRNA biology, underscoring that, while miRNAs are emerging as a highly active and attractive area of research, substantial work remains to fully understand their multifaceted roles and translate them into safe and effective clinical applications.

## 8. Conclusions

MiRNAs play critical roles in fundamental cellular processes, including cell proliferation, differentiation, and apoptosis. More specifically, they are deeply involved in reproductive biology, regulating multiple stages of development, ranging from gametogenesis to pregnancy establishment. Evidence from genetic disruption models further underscores the essential role of miRNAs in regulating normal development and reproductive competence. Despite the considerable progress made in understanding miRNA function, their greatest value may currently lie as research tools rather than immediate clinical applications. Their versatility, abundance, and relative ease of experimental manipulation make them promising candidates for advancing reproductive research. However, the complexity of miRNA regulation, including their dynamic expression patterns, multiple gene targets, and likely interactions with other miRNAs, means that much remains to be understood before they can be reliably applied in livestock production, reproductive technologies, or clinical settings.

**Table 1:** Characteristics and functional roles of selected microRNAs across species.

miRNA	Species	Experimental Approach	Target(s)	Role/Involvement	Reference
**Spermatogenesis**					
let-7a, -7d, -7e and miR-22	Humans	RT-qPCR	Unknown	Increased expression associated with abnormal sperm samples.	[54]
miR-15b	Humans	RT-qPCR	Unknown	Decreased expression associated with abnormal sperm samples.	[54]
miR-34a/b/c	Humans	RT-qPCR	Unknown	Downregulated in infertile men and associated with defective spermatogenesis and male infertility.	[61]
miR-34c-5p	Humans	RT-qPCR and ddPCR	Unknown	Reduced expression is associated with infertility, poorer sperm motility and morphology, and lower probability of obtaining viable embryos following ART.	[61,62,63]
mir-449b-5p	Humans	RT-ddPCR	Unknown	Biomarker associated with sperm DNA fragmentation, sperm concentration, and ART outcomes.	[63]
miR-23a-3p and miR-23b-3p	Humans	RT-qPCR	PFKFB4, HMMR, SPATA6, and TEX15	Negatively associated with sperm count, motility, and morphology through regulation of spermatogenesis-related genes.	[67]
**Oogenesis**					
miR-378	Porcine	Inhibition (Anti-miR miRNA Inhibitor)	CYP19A and progesterone receptor (PGR)	Regulates cumulus cell gene expression and oocyte maturation during COC maturation.	[76]
miR-27a-3p	Ovine	Overexpression (mimics) and Inhibition (inhibitors)	NR5A2	Associated with E2 synthesis suppression in granulosa cells.	[77]
miR-101	Porcine	RT-qPCR and Overexpression (mimics) and Inhibition (inhibitors)	Hyaluronic acid synthase 2 (HAS2)	Regulates oocyte in vitro maturation by modulating cumulus cell expansion.	[79]
miR-31	Porcine	RT-qPCR and Overexpression (mimics) and Inhibition (inhibitors)	MAPK8/JNK pathway	Coordinates cumulus cell expansion and oocyte metabolic reprogramming.	[80]
miR-125a	Porcine	RT-qPCR and Overexpression (mimics) and Inhibition (inhibitors)	Adenosine deaminase RNA (ADAR)	Higher levels appear to disrupt cortical granules and lipid synthesis in oocytes.	[81]
miR-184	Porcine	RT-qPCR	SMAD3	Lower levels are related to granulosa cell apoptosis and follicular atresia, while higher levels possibly promote follicular development and ovarian health.	[84]
miR-143	Ovine	RT-qPCR	CDK2	Regulates follicular dominance and may contribute to sheep fertility by influencing follicular development.	[88]
miR-21	Murine	RT-qPCR and Overexpression (mimics) and Inhibition (miR-off 21)	BMPR2, PTX3, and CDK2AP1	Increased levels in PCOS patients. Overexpression decreases cumulus cell expansion, meiotic progression and GSH levels, while increasing cleavage and blastocyst rates.	[78]
miR-181a	Murine	RT-qPCR	Activin receptor IIA (ACVR2A)	Negatively regulates granulosa cell proliferation and follicular development by inhibiting the activin-Smad2 signalling pathway.	[83]
miR-26b	Porcine	RT-qPCR	Ataxia telangiectasia mutated (ATM)	Promotes granulosa cell apoptosis and follicular atresia through increased DNA breaks.	[86]
miR-330-5p	Murine	RT-qPCR	HDAC3 (ceRNA-mediated regulation via LIPE-AS1)	Regulates oocyte function via the LIPE-AS1/miR-330-5p/HDAC3 axis and may contribute to diminished ovarian reserve (DOR).	[87]
miR-21	Bovine	RT-qPCR	Unknown	Regulates oocyte maturation and subsequent embryo development, potentially through apoptotic/progesterone-signalling pathways.	[101]
**Embryogenesis**					
miR-34c	Murine	RT-qPCR and Inhibition (miRNA inhibitor)	Bcl-2	Downregulation interferes with the first embryonic cell division via modulation of Bcl-2 expression.	[60]
miR-34c	Humans	RT-qPCR and Inhibition (siRNA)	Unknown	Positively correlated with blastocyst rate, quality and pregnancy.	[64]
miR-146b	Bovine	RT-qPCR and Overexpression (mimics) and Inhibition (inhibitors)	Unknown	Regulates embryo development and apoptosis. Overexpression reduces blastocyst quality and development.	[102]
miR-430	Danio rerio (Zebrafish)	Inhibition (locked nucleic acid (LNA)), RT-qPCR, SLAM-seq	Unknown	Regulates EGA and fine-tunes zygotic gene expression during early embryogenesis.	[105]
miR-430	Hybrid fish (Carassius auratus var. (goldfish)) × (Gobiocypris rarus (rare gudgeon))	RT-qPCR and whole-mount in situ (WISH)	Unknown	Regulates maternal-to-zygotic transition (MZT) and embryo viability. Dysregulation is associated with developmental abnormalities and embryo mortality.	[104]
miR-430a	Oryzias latipes (Medaka)	RT-qPCR	Unknown	Expressed after MZT and involved in post-EGA embryonic development.	[106]
miR-200a-5p	Porcine	RT-qPCR	Unknown	Upregulated during EGA timeframe.	[107]
miR-21	Porcine	RT-qPCR and Inhibition (peptide nucleic acids (PNA))	Programmed cell death 4 (PDCD4)	Regulates meiotic maturation and may influence early embryonic development through post-transcriptional regulation of PDCD4.	[108]
miR-155	Porcine	RT-qPCR, Overexpression (mimics) and Inhibition (inhibitors)	Unknown	Regulates embryo developmental competence, stress, and apoptosis.	[112]
miR-17-5p	Bovine	RT-qPCR, Overexpression (mimics) and RNA-seq	TXNIP	Oviductal fluid-derived miRNA that enhances embryonic development to the blastocyst stage.	[122]
**Implantation and Pregnancy**					
miR-124, miR-125, and miR-181	Bovine	Small RNA sequencing (NGS) of EV-derived RNA	Unknown	Enriched in in vivo-derived EVs and associated with implantation and cell lineage specification.	[113]
miR-23b, miR-92a, and miR-409	Bovine	Small RNA sequencing (NGS) of EV-derived RNA	Unknown	Higher expression levels in in vitro-derived EVs and linked to stress and apoptosis pathways.	[113]
miR-124-3p	Murine	RT-qPCR, Overexpression (mimics) and Inhibition (inhibitors)	LIF, MUC1 and BCL2	Negatively regulates uterine receptivity and embryo implantation.	[120]
miR-26a-5p	Porcine	RT-qPCR, Microarrays, and Overexpression (mimics)	Unknown	Involved in promoting trophoblast proliferation and regulates embryo implantation.	[125,126]
miR-125b-5p	Porcine	RT-qPCR, Microarrays, and Overexpression (mimics)	Unknown	Appears to help regulate trophoblast migration and embryo implantation.	[125,126]
miR-143-3p	Porcine	RT-qPCR	Unknown	Potential biomarker of pregnancy.	[124]
miR-125b	Porcine	RT-qPCR	Unknown	Potential biomarker of pregnancy and reproductive status. Abundance correlates with litter size.	[124]
miR-192	Porcine	RT-qPCR	Integrin alpha 4 (ITGA4)	Involved in embryo implantation and pregnancy.	[127]
miR-210	Humans	RT-qPCR, Overexpression (mimics) and Inhibition (morpholino (MO))	Unknown	Regulates trophoblast invasion. Dysregulation is associated with preeclampsia and impaired placental function.	[134]
miR-21	Humans	RT-qPCR	Unknown	Potential biomarker of preeclampsia. Altered expression is associated with disease severity.	[135]
miR-17-3p	Humans	RT-qPCR	Unknown	Associated with preeclampsia and increased oxidative stress.	[136]
miR-214-3p	Murine	RT-qPCR, Overexpression (mimics) and Inhibition (inhibitors)	PlGF and eNOS	Contributes to preeclampsia by impairing trophoblast invasion and endothelial function.	[137]

## Data Availability

No new data were created or analyzed in this study. Data sharing is not applicable to this article.
